# Case-control and family-based association studies of candidate genes in autistic disorder and its endophenotypes: TPH2 and GLO1

**DOI:** 10.1186/1471-2350-8-11

**Published:** 2007-03-08

**Authors:** Roberto Sacco, Veruska Papaleo, Jorg Hager, Francis Rousseau, Rainald Moessner, Roberto Militerni, Carmela Bravaccio, Simona Trillo, Cindy Schneider, Raun Melmed, Maurizio Elia, Paolo Curatolo, Barbara Manzi, Tiziana Pascucci, Stefano Puglisi-Allegra, Karl-Ludvig Reichelt, Antonio M Persico

**Affiliations:** 1 Laboratory of Molecular Psychiatry and Neurogenetics, University "Campus Bio-Medico", Via Longoni 83, I-00155 Rome, Italy; 2 Laboratory of Molecular Psychiatry and Psychiatric Genetics, Department of Experimental Neurosciences, I.R.C.C.S. "Fondazione Santa Lucia", Rome, Italy; 3 IntegraGen SA. Genopole, Evry, France; 4 The Center for Applied Genomics, University of Toronto, Canada; 5 Department of Child Neuropsychiatry, II University of Naples, Italy; 6 Department of Child Neuropsychiatry, University "Federico II", Naples, Italy; 7 ASL RM/B, Rome, Italy; 8 Center for Autism Research and Education, Phoenix, AZ, USA; 9 Southwest Autism Research and Resource Center, Phoenix, AZ, USA; 10 Unit of Neurology and Clinical Neurophysiopathology, I.R.C.C.S. "Oasi Maria S.S.", Troina, EN, Italy; 11 Department of Child Neuropsychiatry, University "Tor Vergata", Rome, Italy; 12 Department of Psychology, University "La Sapienza", Rome, Italy; 13 Laboratory of Behavioral Neurobiology, Department of Experimental Neurosciences, I.R.C.C.S. "Fondazione Santa Lucia", Rome, Italy; 14 Department of Pediatric Research, Rikshospitalet, University of Oslo, Norway

## Abstract

**Background:**

The *TPH2 *gene encodes the enzyme responsible for serotonin (5-HT) synthesis in the Central Nervous System (CNS). Stereotypic and repetitive behaviors are influenced by 5-HT, and initial studies report an association of *TPH2 *alleles with childhood-onset obsessive-compulsive disorder (OCD) and with autism. *GLO1 *encodes glyoxalase I, the enzyme which detoxifies α-oxoaldehydes such as methylglyoxal in all living cells. The A111E GLO1 protein variant, encoded by SNP C419A, was identifed in autopsied autistic brains and proposed to act as an autism susceptibility factor. Hyperserotoninemia, macrocephaly, and peptiduria represent some of the best-characterized endophenotypes in autism research.

**Methods:**

Family-based and case-control association studies were performed on clinical samples drawn from 312 simplex and 29 multiplex families including 371 non-syndromic autistic patients and 156 unaffected siblings, as well as on 171 controls. *TPH2 *SNPs rs4570625 and rs4565946 were genotyped using the TaqMan assay; *GLO1 *SNP C419A was genotyped by PCR and allele-specific restriction digest. Family-based association analyses were performed by TDT and FBAT, case-control by χ^2^, endophenotypic analyses for 5-HT blood levels, cranial circumference and urinary peptide excretion rates by ANOVA and FBAT.

**Results:**

*TPH2 *alleles and haplotypes are not significantly associated in our sample with autism (rs4570625: TDT P = 0.27, and FBAT P = 0.35; rs4565946: TDT P = 0.45, and FBAT P = 0.55; haplotype P = 0.84), with any endophenotype, or with the presence/absence of prominent repetitive and stereotyped behaviors (motor stereotypies: P = 0.81 and 0.84, verbal stereotypies: P = 0.38 and 0.73 for rs4570625 and rs4565946, respectively). Also *GLO1 *alleles display no association with autism (191 patients vs 171 controls, P = 0.36; TDT P = 0.79, and FBAT P = 0.37), but unaffected siblings seemingly carry a protective gene variant marked by the A419 allele (TDT P < 0.05; patients vs unaffected siblings TDT and FBAT P < 0.00001).

**Conclusion:**

*TPH2 *gene variants are unlikely to contribute to autism or to the presence/absence of prominent repetitive behaviors in our sample, although an influence on the intensity of these behaviors in autism cannot be excluded. *GLO1 *gene variants do not confer autism vulnerability in this sample, but allele A419 apparently carries a protective effect, spurring interest into functional correlates of the C419A SNP.

## Background

Autism is a severe disorder of childhood diagnosed on the basis of impaired social interaction and communication, presence of rigid and stereotyped behaviors, and disease onset prior to 3 years of age [1]. Altered prenatal and early-postnatal neurodevelopment plays a pivotal role in autism pathogenesis. Microscopic cytoarchitectonic CNS abnormalities are responsible for the miswiring of neural pathways and for altered information processing, typically in the absence of macroscopic neural malformations or facial dysmorphology [2,3]. Family and twin studies have conclusively demonstrated prominent genetic contributions to autism, with concordance rates of 82–92% in monozygotic twins vs 1–10% in dizygotic twins, sibling recurrence risk at 2–3% vs an incidence of 1–2/1000 in the general population, and heritability estimates above 90% [4,5]. The identification of these genetic underpinnings has proven more complex than anticipated, possibly due to interindividual heterogeneity, epistasis, gene-environment interactions and epigenetic events [6]. In an attempt to pin down single-gene contributions, several investigators have begun selecting candidate genes not merely based on their chromosomal localization, but also by linking their cellular function to specific "endophenotypes" (i.e. heritable clinical, biochemical or morphological traits especially frequent among affected individuals and their first-degree relatives), or by using proteomic studies of post-mortem brain tissue as a starting point. Following these approaches, and under the assumption that single genes may not necessarily yield a detectable signal in linkage studies of polygenic disorders such as autism, *TPH2 *and *GLO1 *were initially assessed despite a relative lack of support by sib-pair analyses for their chromosomal localizations (ch. 12q21.1 and 6p21.3-p21.2, respectively). These initial studies yielded promising evidence supporting *TPH2 *and *GLO1 *roles as autism vulnerability genes [7,8].

Tryptophan hydroxylase-2 (TPH2) is the recently-discovered rate-limiting enzyme for 5-HT synthesis in the CNS, whereas the "classical" TPH isoform, now termed TPH1, is found only in peripheral tissues, except for the pineal gland [9-11]. Several issues contribute to raise interest in *TPH2 *as a candidate gene for autistic disorder: first, 5-HT exerts prominent neurotrophic roles during development, in addition to mediating "classical" neurotransmission [12]; secondly, TPH2 gene variants were found associated with childhood onset OCD [13], and 5-HT influences compulsive and/or stereotyped behaviors to the extent that drugs blocking 5-HT reuptake represent their first-line treatment not only in OCD but also in autism [14]; thirdly, brain imaging studies have revealed that neural responsiveness to emotional stimuli in the amydgala is significantly modulated by TPH2 gene variants [15,16]; finally, one report has provided initial evidence of a possible association between TPH2 gene variants and autism, particularly in patients characterized by more severe repetitive and stereotyped behaviors [7].

Glyoxalase I (GLO1) has been identified using a proteomic approach on post-mortem brain tissues [8]. Gray matter samples from brains of autistic patients display more frequently than unaffected controls a more negatively-charged GLO1 isoform resulting from the single aminoacid substitution A111E, produced by SNP C419A [8]. GLO1 is a cytosolic enzyme responsible for the glutathione-dependent detoxification of α-oxoaldehydes, such as methylglyoxal, spontaneously forming from intermediates of the Embden-Meyerhof glycolytic pathway, such as glyceraldehyde-3-phosphate and dihydroxyacetonephosphate [17]. Interestingly, this initial report also shows reduced GLO1 enzymatic activity in brain extract from autistic patients compared to controls, and accumulation of advanced glycation products resulting from low GLO1 activity [8]. At the genetic level, enhanced frequencies of the A419 allele are found in a small case-control contrast performed on genomic DNA extracted from the same brain tissues of patients and controls [8].

The present study attempts to replicate and extend these findings by using both case-control and family-based association approaches. We also perform quantitative-trait analyses employing some of the best-characterized endophenotypes in autism research, namely cranial circumference, 5-HT blood levels and urinary peptide excretion rates [18,19]. Indeed, macrocephaly (i.e., cranial circumference > 97^th ^percentile) has been consistently found in approximately 20% of autistic patients, 5-HT blood levels are elevated in at least 25% of patients, and excessive peptiduria is found in up to 60% of cases depending on ethnicity and country of origin; sizable familiality and heritability is present for all three parameters ([20-23], and Persico et al, manuscript in preparation for familiality of peptiduria).

## Methods

### Subjects

Families were recruited for this study based on the presence of a proband diagnosed with primary autistic disorder (i.e., idiopathic, non-syndromic autism). The composition of our clinical sample is summarized in Table 1. Out of this global sample, 210 Italian families and 24 Caucasian-American families from Arizona were involved in the TPH2 study, whereas GLO1 was genotyped in a partly-overlapping set including 176 Italian families and all 76 Caucasian-American families available. Demographic and clinical characteristics, as well as inclusion criteria, and diagnostic screening methods used to exclude probands with syndromic autism have been previously reported [18]. Briefly, patients fulfilling DSM-IV diagnostic criteria for Autistic Disorder [1] were screened for non-syndromic autism using MRI, EEG, audiometry, urinary aminoacid and organic acid measurements, cytogenetic and fragile-X testing. Patients with dysmorphic features were excluded even in the absence of detectable cytogenetic alterations. Patients with sporadic seizures (i.e., < 1 every 6 months) were included; patients with frequent seizures or focal neurological deficits were excluded. Autistic behaviors were assessed using the official Italian version of the Autism Diagnostic Observation Schedule (ADOS) and the Autism Diagnostic Interview-Revised (ADI-R) [24,25]; adaptive functioning was assessed using the Vineland Adaptive Behavior Scales (VABS); I.Q. was determined using either the Griffith Mental Developmental Scales, the Coloured Raven Matrices, the Bayley Developmental Scales or the Leiter International Performance Scale. In addition, a total of 156 unaffected siblings was also assessed, including 128 siblings from simplex families and 28 from multiplex families. All parents gave written informed consent for themselves and for their children, using the consent form approved by the I.R.B. of U.C.B.M. (Rome, Italy). Finally, we assessed 180 unaffected Italian controls, including 166 individuals whose blood was drawn at the Laboratory of the "S. Cuore" Clinic (Rome, Italy), as prescribed by family practitioners for a broad range of physical complaints unrelated to psychiatric disorders, and 14 medical and nursing students recruited at U.C.B.M. (Rome, Italy).

**Table 1:** Composition of the complete sample.

		*Number of Families*		*Number of Trios*	
		
*Site*	*Number of Individuals with Autism*	*Simplex*	*Multiplex*	*Complete*	*Incomplete*
II University of Naples (Naples, Italy)	121	121	-	107	14
I.R.C.C.S. "Ospedale Bambino-Gesù" (Rome, Italy)	42	42	-	38	4
I.R.C.C.S. "Oasi Maria S.S." (Troina, Italy)	42	40	1	41	1
U.C.B.M. (Rome, Italy)	21	21	-	21	-
II University of Rome (Rome, Italy)	22	20	1	21	-
University of Milan (Milan, Italy)	15	15	-	15	-
University of Turin (Turin, Italy)	4	4	-	4	-
**Italian Families**	***267***	***263***	***2***	***247***	***19***

A.G.R.E. Consortium	60	15	23*	38	-
Southwest Autism Research Center (Phoenix, AZ)	44	34	4	38	-
**Caucasian-American Families**	***104***	***49***	***27***	***76***	***-***

**Total Sample**	***371***	***312***	***29***	***323***	***19***

*DNA was not available for one of the two affected children from one multiplex family from the A.G.R.E. Consortium. This family is still listed as "multiplex" in the table.

### Markers and genotyping

SNPs rs4570625 and rs4565946 are located in the putative transcriptional control region [G(-703)T] and in intron 2 of the *TPH2 *gene, respectively [13]. These SNPs were selected because their G-C haplotype is preferentially transmitted to children and adolescents with OCD [13], and due to evidence of functional correlates with neural activity, at least for alleles at SNP rs4570625 [15,16]. SNPs rs4570625 and rs4565946 were genotyped using the TaqMan method (Applied Biosystems, Foster City, CA), according to the manufacturer's guidelines. The following primers were used:

rs4570625

forward primer: ACACTCACACATTTGCATGCAC

reverse primer: CATTGACCAACTCCATTTTATGTTAATAAGCT

reporter 1 (VIC) sequence: CTTGACATATTCTAATTTT

reporter 2 (FAM) sequence: ACTTGACATATTATAATTTT

rs4565946:

forward primer: TCACTCTGCCATCAGCTAGTCA

reverse primer: CTGAGGTCCAGATGGGTTAAATGG

reporter 1(VIC) sequence: CTTAGCTAAGGCCCCG

reporter 2 (FAM) sequence: CTTAGCTAAGACCCCG

The PCR amplification protocol for the TaqMan assays includes denaturation at 95°C for 10 min, followed by 40 cycles at 92°C for 15 sec, 60°C for 1 min, and 72°C for 45 sec, followed by elongation at 72°C for 5 min. The TaqMan assays were then read on a 7900HT Fast Real-Time PCR System and alleles were called using the SDS software (Applied Biosystems, Foster City, CA).

The *GLO1 *SNP C419A was genotyped by PCR amplification of genomic DNA (80 ng) using the following primers GLO1-F [5'-TGTCCAGTACCTGTATTTACTG-3'] and GLO1-R [5'-GCAAACTTACCGAATCCTCG-3']. The PCR amplification protocol includes denaturation at 95°C for 3 min, followed by 35 cycles at 95°C for 30 sec, 57°C for 30 sec, and 72°C for 45 sec, followed by elongation at 72°C for 5 min. The PCR product was cut with BSM*AI *(5 U) at 55°C overnight and run in 2% agarose or 5% acrylamide gels, yielding different restriction patterns for the C allele (259 bp + 36 bp) and the A allele (198 bp + 61 bp + 36 bp). An alternative restriction pattern was also identified (C = 295 bp, A = 234 bp + 61 bp) in 3/171 (1.8%) controls, as well as in one father and in one unaffected sibling.

### Endophenotype measures

Blood samples for 5-HT levels were obtained from all family members and centrifuged within 20 min of venipuncture at 140 g for 25 min at 4°C; 1 ml of supernatant (i.e., platelet-rich plasma) was stored at -80°C and assessed by HPLC, as described [22]. Urinary peptide excretion analysis was performed by HPLC on the first morning urine samples of all family members, as described [23]. The total area of peaks under the 215 nm absorption curve (AUC) in the peptide region following the hippuric acid peak was calculated and expressed in μm^2^. Head circumference was measured in autistic patients and unaffected siblings by trained physicians using a non-stretchable plastic measuring tape graded in millimeters, placed over the maximum fronto-occipital head perimeter [18].

### Data analysis

Hardy-Weinberg analyses were performed using the HWE program of the LINKUTIL software package [26]. Case-control allelic and genotypic distributions were contrasted using the χ^2 ^statistics, following randomized selection of one patient per multiplex family. Single-marker and haplotype family-based association analyses were performed applying the transmission/disequilibrium test (TDT) [27] using the TDTPHASE software of the UNPHASED package [28]; only complete trios and one trio per multiplex family were included in these analyses. Furthermore, family-based association analyses were also performed using the FBAT software, which emphasizes contrasts between siblings allowing to fully use the genetic information provided by multiplex families [29,30]. All family-based association analyses were carried out on Italian and Caucasian-American families merged together, after population structure analyses provided no evidence of genetic dyshomogeneity in a subgroup of 179 autistic patients including 155 Italians and 24 Caucasian-Americans individuals randomly chosen one per family, genotyped at ninety unlinked SNPs distributed genome-wide and analyzed using the STRUCTURE program [31]. Since these stratification analysis did not include unaffected controls, case-control contrasts employed only individuals of Italian ancestry. Data are expressed as mean ± S.E.M., except for head circumference and urinary peptide excretion rates, expressed as median ± semi-interquartilic range (I.Q.R.). Head circumference measures were transformed into percentiles using sex- and age-specific standard tables [32,33]. Two-tail P values are reported.

## Results

### TPH2

Both rs4570625 and rs4565946 are in Hardy-Weinberg equilibrium in autistic patients and in their first-degree relatives (data not shown). The two SNPs are in strong linkage disequilibrium (D' = 0.91, P < 0.001), as previously found in other samples [13]. Both TDT and FBAT analyses, presented in table 2, provide no evidence of significant association with autism either for single markers, or for two-marker haplotypes (global P-value for haplotype analysis = 0.84). Single-marker FBAT analyses show no trend towards possible associations with endophenotypic quantitative traits including cranial circumference (rs4570625: P = 0.16; rs4565946: P = 0.13), and urinary peptide excretion rates (rs4570625: P = 0.16; rs4565946: P = 0.52)(table 3). A non-significant trend towards higher 5-HT blood levels is seen in patients carrying TPH2 alleles G and C at rs4570625 and rs4565946, respectively (table 3) (rs4570625: P = 0.0504; rs4565946: P = 0.69). Finally, *TPH2 *gene variants marked by these two SNPs also do not seem to influence whether at the time of clinical intake autistic patients display prominent motor or verbal stereotypies (table 4). Identically negative outcomes are also obtained performing single-marker FBAT analyses on subsets of families selected for probands displaying motor stereotypies (rs4570625: N = 66, P = 0.58; rs4565946: N = 77, P = 0.92), no motor stereotypies (rs4570625: N = 37, P = 0.75; rs4565946: N = 35, P = 0.61), or with verbal/vocal stereotypies (rs4570625: N = 34, P = 0.43; rs4565946: N = 47, P = 0.53), or no verbal/vocal stereotypies (rs4570625: N = 75, P = 0.69; rs4565946: N = 73, P = 0.77).

**Table 2:** TDT and FBAT analyses of SNPs rs4570625 and rs4565946, and relative haplotypes at the TPH2 locus.*

***TDT***	***TPH2 – RS4570625***		***TPH2 – rs4565946***			***HAPLOTYPES RS4570625-RS4565946***				
	***N = 224 complete trios***		***N = 219 complete trios***			***Haplotype***	***T***	***NT***	***χ^2 ^(1df)***	***P-value***
*G Transmitted*	88	χ^2 ^= 1.21(1 df), P = 0.27	*C Transmitted*	107	χ^2 ^= 0.57(1 df), P = 0.45	G-C	73	72	0.01	0.92
*T Transmitted*	77		*T Transmitted*	116		G-T	87	74	1.05	0.31
						T-C	51	63	1.26	0.26
						T-T	1	3	1.00	0.32

***FBAT***	**Allele**	**N. of families**	**S**	**E(S)**	**Var(S)**	**Z**	**P**			

**rs4570625**	G	136	194.000	188.000	42.000	0.926	0.35			
	T	136	84.000	90.000	42.000	-0.926				
**rs4565946**	C	166	161.000	165.500	57.750	-0.592	0.55			
	T	166	183.000	178.500	57.750	0.592				
**Haplotypes (Estimated Freq)**										
G-T (0.486)		160.9	195.112	190.637	56.686	0.594	0.55			
G-C (0.296)		154.0	127.888	126.863	48.312	0.147	0.88			
T-C (0.211)		125.4	78.112	83.137	39.218	-0.802	0.42			
T-T (0.008)		6.7	-	-	-	-	-			

*FBAT analyses are performed under an additive model [29,30]. P-values are rounded to the second digit.

**Table 3:** Serotonin blood levels, urinary peptide excretion rates, and head circumference by TPH2 and GLO1 genotypes. *

**TPH2**	**AUTISTIC PATIENTS – RS4570625**				**AUTISTIC PATIENTS – RS4565946**			
	**GG**	**GT**	**TT**	**Statistics**	**CC**	**CT**	**TT**	**Statistics**

Serotonin Blood Levels (ng/ml)	352.4 ± 35.7 *(90)*	293.8 ± 40.7 *(39)*	261.2 ± 57.0 *(6)*	F = 0.642 (2,132 df), P = 0.53	377.4 ± 64.9 *(42)*	328.2 ± 33.2 *(58)*	289.1 ± 42.1 *(32)*	F = 0.742 (2,129 df), P = 0.48
Urinary Peptides (AUC in μm^2^)	309.5 ± 122 *(80)*	297.5 ± 97.5 *(42)*	332.0 *(2)*	K-W χ^2 ^= 3.340, 2 df, P = 0.19	306.0 ± 127.5 *(33)*	298.0 ± 86.5 *(57)*	296.0 ± 150 *(32)*	K-W χ^2 ^= 0.172, 2 df, P = 0.92
Head Circumference (percentile)	82.5 ± 23.7 *(97)*	82.5 ± 17.5 *(57)*	50.0 ± 24 *(6)*	K-W χ^2 ^= 2.324, 2 df, P = 0.31	75.0 ± 23.7 *(42)*	75.0 ± 23.7 *(72)*	90.0 ± 23.7 *(42)*	K-W χ^2 ^= 0.958, 2 df, P = 0.62

**GLO1**	**AUTISTIC PATIENTS**				**UNAFFECTED SIBLINGS**			

	**AA**	**AC**	**CC**	**Statistics**	**AA**	**AC**	**CC**	**Statistics**

Serotonin Blood Levels (ng/ml)	213.2 ± 22.5 *(49)*	208.5 ± 11.7 *(76)*	244.3 ± 24.2 *(29)*	F = 0.857 (2,153 df), P = 0.43	201.0 ± 33.5 *(22)*	189.8 ± 29.4 *(18)*	177.8 ± 20.9 *(10)*	F = 0.111 (2,49 df), P = 0.90
Urinary Peptides (AUC in μm^2^)	324.0 ± 110 *(55)*	313.0 ± 120 *(85)*	319.0 ± 132 *(37)*	K-W χ^2 ^= 0.711, 2 df, P = 0.70	212.5 ± 65 *(26)*	196.0 ± 98 *(25)*	290.0 ± 146.5 *(15)*	K-W χ^2 ^= 1.363, 2 df, P = 0.51
Head Circumference (percentile)	82.5 ± 17.5 *(63)*	82.5 ± 23.8 *(99)*	75.0 ± 33.2 *(40)*	K-W χ^2 ^= 1.325, 2 df, P = 0.52	86.3 ± 19.1 *(22)*	82.5 ± 23.8 *(22)*	82.5 ± 31.6 *(10)*	K-W χ^2 ^= 0.788, 2 df, P = 0.67

* Data are expressed as mean ± S.E.M. for serotonin blood levels, and median ± semi-interquartilic range for amounts of urinary peptides and head circumference; 2-tail P-values are reported. Sample sizes are shown in italics; K-W = Kruskal-Wallis non-parametric ANOVA.

**Table 4:** TPH2 alleles in autistic patients with(+) or without (-) motor stereotypies (MS) or verbal stereotypies (VS).

**SNP**	**Allele**	**AUTISTIC PATIENTS**				CONTROLS*
			
		M.S. PRESENT	M.S. ABSENT	V.S. PRESENT	V.S. ABSENT	
**rs4570625**	**G**	104/148 (70.3%)	37/54 (68.5%)	60/82 (73.2%)	74/110 (67.3%)	206/268 (76.9%)
	**T**	44/148 (29.7%)	17/54 (31.5%)	22/82 (26.8%)	36/110 (32.7%)	62/268 (23.1%)
	**statistics**	M.S. + vs -: χ^2 ^= 0.06, 1 df, P = 0.81		V.S. + vs -: χ^2 ^= 0.78 1 df, P = 0.38		
		M.S. + vs Controls: χ^2 ^= 2.18, 1 df, P = 0.14		V.S. + vs Controls:χ^2 ^= 0.47, 1 df, P = 0.49		
		M.S. - vs Controls: χ^2 ^= 1.69, 1 df, P = 0.19		V.S. - vs Controls:χ^2 ^= 3.74, 1 df, P = 0.053		

**rs4565946**	**C**	79/153 (51.6%)	34/64 (53.1%)	51/95 (53.7%)	58/113 (51.3%)	156/268 (58.2%)
	**T**	74/153 (48.4%)	30/64 (46.9%)	44/95 (46.3%)	55/113 (48.7%)	112/268 (41.8%)
	**statistics**	M.S. + vs -: χ^2 ^= 0.04 1 df, P = 0.84		V.S. + vs -: χ^2 ^= 0.11, 1 df, P = 0.73		
		M.S. + vs Controls: χ^2 ^= 1.71, 1 df, P = 0.19		V.S. + vs Controls: χ^2 ^= 0.59, 1 df, P = 0.44		
		M.S. - vs Controls: χ^2 ^= 0.55, 1 df, P = 0.46		V.S. - vs Controls: χ^2 ^= 1.53, 1 df, P = 0.22		

**Haplotypes**		Estimated Frequency in M.S. and/or V.S. present		Estimated Frequency in M.S. and/or V.S. absent		
**G-C**		0.3271		0.3065		
**G-T**		0.458		0.4516		
**T-C**		0.201		0.2419		
**T-T**		0.014		-		

Likelihood ratio test: null = -232.5, alternative = -232, LRS = 1.034, 3 df, P = 0.59						

*Allelic frequencies for controls are from ref 13.

### GLO1

The *GLO1 *C419A SNP is in Hardy-Weinberg equilibrium in autistic patients and in their first-degree relatives (data not shown). Case-control, TDT and FBAT analyses provide no evidence of association between C419A alleles and autism (table 5). Furthermore, there is no trend towards an association with serotonin blood levels (informative families N = 93, FBAT P = 0.17), cranial circumference (N = 96, P = 0.43), and urinary peptide excretion rates (N = 115, P = 0.96) (table 3). However, TDT analyses support the existence of a protective A419 variant that is preferentially transmitted to unaffected siblings, particularly from the paternal side (table 5). Single-marker FBAT analyses display only a non-significant trend in this direction, possibly due to small sample size, but confirm a highly significant divergence in allelic transmission probabilities between autistic patients and unaffected siblings at this locus (P < 1 × 10^-5^, table 5).

**Table 5:** Case-control, TDT and FBAT (additive model) analyses of SNP C419A (A111E) at the GLO1 locus.

***CASE-CONTROL***	ITALIAN PATIENTS (N = 191)	ITALIAN CONTROLS (N = 171)	U.S. PATIENTS (N = 101)			ITALIAN PATIENTS (chr N = 382)	ITALIAN CONTROLS (chr N = 342)	U.S. PATIENTS (chr N = 202)
**A/A**	67 (35.1%)	52 (30.4%)	34 (33.7%)		**A**	221 (.5785)	186 (.5439)	115 (.5693)
**A/C**	87 (45.5%)	82 (48.0%)	47 (46.5%)		**C**	161 (.4215)	156 (.4561)	87 (.4307)
**C/C**	37 (19.4%)	37 (21.6%)	20 (19.8%)					

	Italian patient vs control genotypes χ^2 ^= 0.94, 2 df, P = 0.63				Italian patient vs control alleles χ^2 ^= 0.88, 1 df, P = 0.36			

***TDT***	**Autistic Patients**		**Unaffected Siblings**					
			
	***N = 236 complete trios***		***N = 100 complete trios***		Maternal transmissions		Paternal transmissions	

*A Transmitted*	105	χ^2 ^= 0.074(1df), P = 0.79	56	χ^2 ^= 4.35(1df), **P < 0.05**	21	χ^2 ^= 1.00(1df), P = 0.32	26	χ^2 ^= 5.16(1df), **P < 0.05**
*C Transmitted*	109		36		15		12	

TDT autistic patients vs unaffected siblings: χ^2 ^= 24.48 (1df), **P < 0.000001**								

***FBAT***		**Allele**	**N. of families**	**S**	**E(S)**	**Var(S)**	**Z**	**P**

**Autistic Patients**		A	170	199.000	206.000	61.500	-0.893	0.37
		C	170	189.000	182.000	61.500	0.893	
**Unaffected Siblings**		A	77	111.000	103.000	30.000	1.461	0.14
		C	77	75.000	83.000	30.000	-1.461	

FBAT autistic patients vs unaffected siblings: Z = 17.44, **P < 0.00001**								

## Discussion

The present study provides evidence that *TPH2 *and *GLO1 *gene variants do not contribute significantly to autism pathogenesis in our sample, while *GLO1 *could seemingly exert a protective effect in unaffected siblings. Our conclusions are strengthened by the consistency of multiple statistical approaches and by the parallel assessment of some of the most reliable biochemical and morphological endophenotypes described in autism research to this date, namely macrocephaly, hyperserotoninemia and enhanced peptiduria [20-23]. These heritable traits are believed to be more directly linked to the genetic make-up of an individual than complex clinical symptoms, and could thus possibly identify subgroups of autistic patients characterized by relatively homogenous pathogenetic processes. These parameters were thus chosen on this ground, and not following *a-priori *hypotheses. In particular, 5-HT blood levels do not reflect TPH2 activity, as peripheral 5-HT largely comes from the digestive tract and is thus produced by TPH1 [9-11]. Therefore, the non-significant trend towards an association between TPH2 gene variants and 5-HT blood levels either represents a spurious finding, or necessarily stems from an indirect pathophysiological link which will require further investigation.

Some differences must be drawn in the interpretation of the data pertaining to *TPH2 *and *GLO1*. Our results exclude causal TPH2 roles in our sample, and do not support the hypothesis that TPH2 gene variants may exert a major influence on repetitive and stereotypic behaviors in autistic patients. This conclusion confirms the outcome of a recent study, reporting no association either with autism, or with obsessive-compulsive and self-stimulatory behaviors in autistic patients [34]. Collectively, these findings, although negative, are important due to the relevant roles played by 5-HT during neurodevelopment [12], and because they help us interpret contributions to autism by serotoninergic genes. TPH2 and the 5-HT transporter (5-HTT) play pivotal roles in central serotoninergic neurotransmission. TPH2 determines the rate of 5-HT biosynthesis in the CNS [9-11], whereas the 5-HTT terminates the action of extracellular 5-HT on its receptors through reuptake [35]. During neurodevelopment, 5-HT exerts impressive neurotrophic effects on cell proliferation, differentiation, and migration, programmed cell death, cell-cell coupling, synaptogenesis and cytoskeletal plasticity [12]. Many of these processes appear altered in neuropathological studies of autistic brains [2,3]. Furthermore, a recent quantitative brain imaging study has revealed increased cortical gray matter volumes in autistic patients carrying 5-HTT gene variants yielding slower extracellular 5-HT clearance rates, resulting in higher extracellular 5-HT levels [36]. This finding parallels results obtained in 5-HTT knockout mice backcrossed into C57BL/6J, displaying increased neuronal cell density and thickness of supragranular and infragranular neocortical layers compared to wild-type mice [37]. In contrast to these neurobiological data, strongly supporting the trophic effects exerted by elevated extracellular 5-HT levels in autism, results of genetic studies on serotoninergic gene variants in autism are mostly negative or mixed at best. Indeed, the present and prior studies jointly indicate that (a) in the vast majority of cases, functional serotoninergic gene variants play a modulatory, but not a causal role in autism; (b) 5-HT synthesis does not contribute to produce increased levels and/or prolonged persistence of extracellular 5-HT, which largely stems from reduced 5-HT uptake and could possibly play a role in eccessive neurotrophism and macrocephaly in autism; (c) 5-HT could modulate not only morphological features such as head circumference, but also repetitive and stereotypic behaviours in autism, as well as in OCD [7,13,14,38]. Unfortunately the approved Italian translation of the Autism Diagnostic Interview-Revised (ADI-R) has become available only recently [25], and this instrument was not available when the vast majority of our sample was recruited. However, the presence/absence of relevant verbal and motor stereotypic behaviors that was assessed here by an experienced clinician at intake largely corresponds to the current presence of "stereotyped utterances and delayed echolalia", "hand and finger mannerisms", "other complex mannerisms or stereotyped body movements", and "midline hand movements", as assessed by ADI-R items n. 33, 77, 78, and 79, respectively [25]. Therefore, despite representing a crude, single time-point measure, it allows us to exclude prominent roles for TPH2 gene variants in these behavioural symptoms. Searches for modulatory effects on repetitive and stereotypic behaviors will require finer, possibly longitudinal measures.

On the other hand, GLO1 allele A419 seemingly exerts a protective effect among unaffected siblings, whose allelic transmission patterns differ significantly from those of their autistic siblings, both using TDT and FBAT. The discrepancy between initial findings by Junaid and Colleagues [8], proposing A419 as an autism vulnerability allele, and our findings may stem from at least two potential sources. First, it is important to notice that the A419 allele frequencies found by Junaid and Colleagues [8] in autistic patients are very similar to those reported here (0.6056 vs 0.5785 and 0.5693 in our Italian and Caucasian-American patients, respectively), whereas their control allele frequencies are much lower than those found in our controls (0.4400 vs 0.5439). It is likely that a small sample size including only 50 controls, encompassing both normal subjects and patients with Batten disease or fragile-X syndrome instead of randomly chosen unaffected individuals, may have diminished the reliability of allele frequency estimates in the general population, skewing case-control statistics [8]. The definition of A419 as a risk allele for autism would thus not seem fully justified already on the basis of the genetic data set presented in the initial study [8]. Secondly, the present study is the first one including also unaffected siblings, which were not assessed previously [8]. The existence of significant differences in allelic transmission rates between our autistic patients and unaffected siblings does point towards the existence of functional GLO1 gene variants, deserving further characterization in order to understand their protective role in families with an autistic proband. These variants may be represented by the A419 allele itself, but could also consist in polymorphisms located in the transcriptional regulatory regions of the GLO1 gene and in linkage disequilibrium with the C419A SNP. This possibility is consistent with the decreased glyoxalase I enzymatic activity found in post-mortem neocortical tissues of autistic patients, as compared to controls [8].

## Conclusion

Based on the consistency between case-control and family-based association analyses, we can exclude in our sample a major role for TPH2 gene variants in autism pathogenesis, and in determining the presence of prominent repetitive and stereotypic behaviors assessed at the time of patient recruitment by an experienced clinician. Our study cannot exclude that functional TPH2 gene variants may influence the intensity and course of repetitive and stereotypic behaviors in the longer term. Also GLO1 protein variants encoded by the C419A SNP seemingly do not confer autism vulnerability in this sample, but allele A419 apparently carries a protective effect among unaffected siblings, spurring further interest into the functional correlates of the C419A SNP and of other polymorphisms located in transcriptional regulatory regions of this gene.

## List of abbreviations

5-HT, serotonin; 5-HTT, serotonin transporter; CNS, central nervous system; FBAT, family-based association test; GLO1, glyoxalase I; K-W, Kruskal-Wallis non parametric ANOVA; MS, motor stereotypies; OCD, obsessive-compulsive disorder; SNP, single nucleotide polymorphism; TDT, transmission/disequilibrium test; TPH2, tryptophan hydroxylase-2; VS, verbal stereotypies.

## Competing interests

The author(s) declare that they have no competing interests.

## Authors' contributions

RS participated in study design and performed the statistical analyses, VP carried out GLO1 genotypings, JH and FR performed TPH2 genotypings and commented on the manuscript, RMoe participated in study design for TPH2. Patient recruitment, clinical assessment and sample collections were performed by RMi and CB in Naples, PC, BM and ST in Rome, CS and RMe in Phoenix, ME in Troina. TP and SPA measured 5-HT blood levels, KLR determined urinary peptide excretion rates, and AMP contributed to study design, coordinated recruitment and data collection, and drafted the manuscript. Authors read and approved the final manuscript.

## Pre-publication history

The pre-publication history for this paper can be accessed here:


